# Personal

**Published:** 1904-12

**Authors:** 


### PERSONAL.

Dr. Lawrence Hendee, of Buffalo, has returned from a year's study in Germany, and has begun practice in this city; his office being at 523 Franklin street. Hours: 8 to 9.30 and 1.30 to 3. Telephone, Tupper 1340-Y.

---

Dr. William Japp Sinclair, professor of obstetrics and gynecology in Victoria University, Manchester, was the recipient of the honor of knighthood, conferred by King Edward upon his recent birthday. The honor was given on account of valuable national services in medical science. Professor Sinclair is a brother of Angus Sinclair, publisher of *The Automobile Magazine* and other journals of New York, and a prominent member of the Automobile Club of America. Sir William is an Honorary Fellow of the American Association of Obstetricians and Gynecologists. He is a frequent visitor to American shores where he is always welcome.

---

Dr. W. S. Renner, of Buffalo, attended the dinner given by New York laryngologists to Sir Felix Semon at Delmonico's, Tuesday evening, November 1, 1904. More than one hundred covers were laid and the participants included members of the section on laryngology of the New York Academy of Medicine and of the American Laryngological Association, as well as a few specially invited guests.

---

Dr. Frederick H. Millener, of Buffalo, has removed his office from 724 Main street to 139 North Pearl street. His hours remain unchanged.

---

Dr. W. E. Dignen, of Buffalo, announces the removal of his office from 544 to 490 Plymouth avenue, corner of Massachusetts and Plymouth avenues. Hours: 8 to 10 a. m., 1 to 3 and 7 to 8 p. m. Both telephones.

---

Dr. Frederick J. Barrett, of Buffalo, has opened an office at 305 Pearl, corner of Mohawk street, where he will limit his practice to the treatment of diseases of the eye and ear. Hours: 9 to 1 and 4 to 5 p. m. Sundays by appointment. Residence: 290 Breckenridge street. Hours: 7 p. m., and by appointment.

---

Dr. Arthur R. Bradbury, U. of B. 1892, lately of Grand Island, has reëstablished himself in the practice of medicine at Buffalo. His office and residence are at 142 Ontario street. He was for several years house surgeon at the Fitch Accident Hospital.

---

Drs. F. W. and James T. Jelks, of Hot Springs, Ark., announce their removal to offices in the Dugan-Stuart Building, opposite the Arlington Hotel. New telephone, 512.

